# Effect of Tenapanor on Phosphate Binder Pill Burden in Hemodialysis Patients

**DOI:** 10.1016/j.ekir.2021.06.030

**Published:** 2021-07-08

**Authors:** Tadao Akizawa, Yu Sato, Kazuaki Ikejiri, Hironori Kanda, Masafumi Fukagawa

**Affiliations:** 1 Division of Nephrology, Department of Medicine, Showa University School of Medicine, Tokyo, Japan; 2 R&D Division, Kyowa Kirin Co., Ltd., Tokyo, Japan; 3 Division of Nephrology, Endocrinology, and Metabolism, Department of Internal Medicine, Tokai University School of Medicine, Kanagawa, Japan

**Keywords:** chronic kidney disease, hyperphosphatemia, phosphate binder, pill burden, sodium/hydrogen exchanger isoform 3, tenapanor

## Abstract

**Introduction:**

The current management of hyperphosphatemia with phosphate binders is associated with insufficient phosphorus control and a significant pill burden. Tenapanor, a first-in-class, phosphate absorption inhibitor, is expected to control phosphorus and decrease pill burden because of its small pill size and twice daily dosing regimen. This study evaluated tenapanor effectiveness on reducing the phosphate binder pill burden during a 26-week treatment period in Japanese hemodialysis patients.

**Methods:**

In this multicenter, open-label, single-arm study, hemodialysis patients whose serum phosphorus level was 3.5 to 7.0 mg/dl received tenapanor 30 mg twice daily orally added to their phosphate binder regimen. The phosphate binder dosage was adjusted to achieve a serum phosphorus level within the baseline range of ±0.5 mg/dl. The primary end point was the percentage of patients who achieved a ≥30% decrease in the number of phosphate binders and tenapanor tablets prescribed daily compared with the number of phosphate binder tablets at baseline.

**Results:**

Of the 67 patients enrolled, 43 completed the study. At baseline, the mean total number of phosphate binder tablets per day was 14.7, which decreased to 3.0 tablets per day at week 26. The primary end point was achieved in 71.6% of patients (*P* < 0.001). The phosphate binder was completely switched to tenapanor in 28.4% of patients (*P* < 0.001). The mean phosphorus levels were relatively well controlled (5.19 and 4.71 mg/dl at baseline and week 26, respectively). The most frequent drug-related adverse event (AE) was diarrhea (74.6%).

**Conclusion:**

Tenapanor provided effective phosphorus control and decreased the number of phosphate binder tablets. The management of drug-related diarrhea will facilitate more widespread use of tenapanor.

## Graphical abstract

**Figure:**
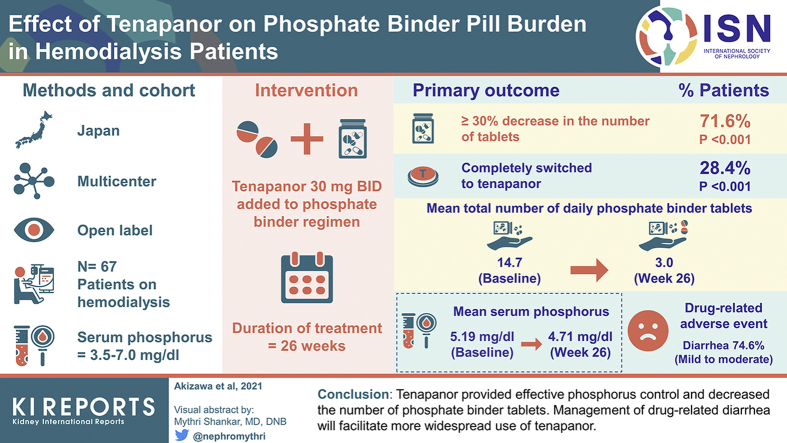

The treatment of hyperphosphatemia to achieve and maintain serum phosphorus levels within the target range can help prevent bone disease, vascular calcifications, and cardiovascular disease.^1^^,^^2^ If dietary interventions and dialysis fail to manage serum phosphorus levels for hemodialysis patients properly, drug therapy with phosphate binders is the recommended treatment approach.^3^, ^4^, ^5^, ^6^

Phosphate binders act by binding to phosphoric acid in the gastrointestinal tract and promoting phosphorus excretion into the feces.^7^ However, some phosphate binders lead to adverse drug reactions,^8^ including iron deposition in organs, hypercalcemia, and gastrointestinal symptoms (e.g., diarrhea and changes in stool color). Additionally, adherence to treatment is another major issue with phosphate binder treatment. These drugs need to be taken before or after every meal, and tablets are usually large and require many pills per dose; thus, this treatment results in a substantial pill burden.^9^^,^^10^ These factors have a detrimental effect on drug adherence. Therefore, a drug that can contribute to the control of serum phosphorus levels and that can resolve these issues can help improve patient adherence to medications and has the potential to improve the overall ability to effectively control phosphorus.

Hemodialysis patients tend to have other comorbidities, such as hypertension, diabetes, cardiovascular disease, and mineral and bone disorder. Thus, patients must also face the pill burden related to the treatment of these comorbidities. It has been estimated that dialysis patients are prescribed 10 to 12 different medications and take an average of 20 pills per day,^11^ of which half are presumed to be phosphate binders.

Tenapanor is a first-in-class, nonbinder, phosphate absorption inhibitor that reduces phosphate absorption by local inhibition of the sodium/hydrogen exchanger isoform 3. This results in reduced sodium absorption and, consequently, proton retention in cells. This modest intracellular proton retention induces a conformational change in tight junctions, reducing permeability specific to phosphate, which results in decreased phosphate absorption through the paracellular pathway and a decrease in serum phosphorus levels.^12^ Thus, tenapanor’s mechanism of action is different from that of conventional phosphate binders. Tenapanor was designed to work in the gastrointestinal tract and is minimally absorbed. It is a calcium-free, nonmetal, nonpolymeric agent. Additionally, tenapanor is expected to decrease the phosphate binder pill burden or eliminate the need for phosphate binder pills because the dose consists of 1 small tablet taken twice daily.

The serum phosphorus level–lowering effect and safety profile of tenapanor have not been confirmed in Japanese hemodialysis patients. The effects of adding tenapanor to the existing phosphate binder regimen remain to be elucidated. This study aimed to confirm the serum phosphorus level–controlling effect and investigate the safety profile of tenapanor and whether tenapanor reduced the phosphate binder pill burden for these patients.

## Materials and Methods

### Study Design and Treatment

**Figure 1:**
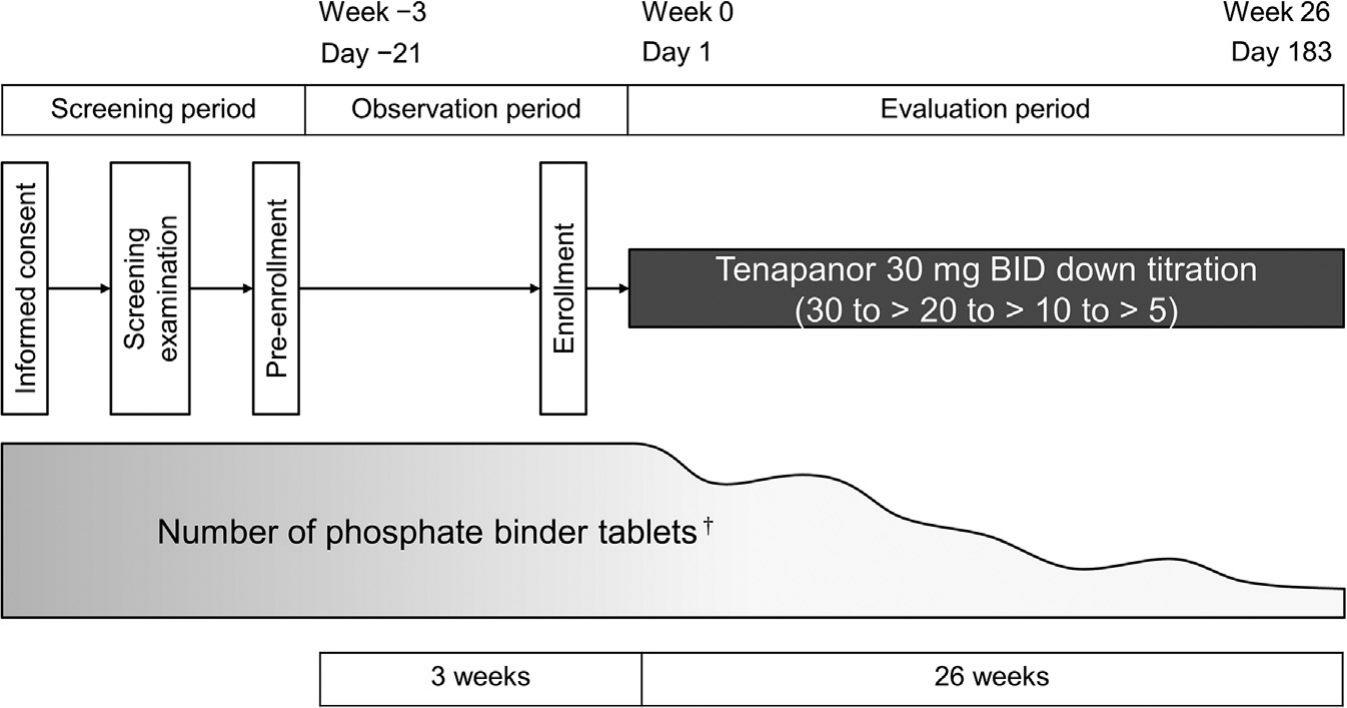
Study design. ^†^Including all existing phosphate binders. BID, twice daily.

This study (Clinicaltrials.gov identifier: NCT03831607) was a multicenter, open-label, single-arm study conducted between December 2018 and November 2019 at 8 institutions in Japan. It was composed of a screening period, a 3-week observation period, and a 26-week treatment period (Figure 1). During the observation and treatment periods, laboratory tests were conducted every week on the date of dialysis after the longest dialysis interval. At week −3, 0, 8, and 25, laboratory tests were also conducted before the first short dialysis interval.

Patients received oral tenapanor 30 mg twice daily immediately before meals (breakfast and dinner). Up to week 8, the tenapanor dose could be decreased in a stepwise manner (from 30 mg to 20 mg, 10 mg, or 5 mg) for patients with gastrointestinal-related AEs for which a causal relationship could not be excluded based on the investigators’ judgment. The dose could also be decreased if the serum phosphorus level decreased below 2.5 mg/dl after the patient completely switched from the phosphate binder. The dose was reduced from week 9 onward if AEs occurred for which a causal relationship with the study drug could not be excluded.

In this study, patients were prescribed 6 types of phosphate binders (Table 1). Patients continued their individual dose regimens at pre-enrollment. The dose of phosphate binders was adjusted according to the investigators’ judgment, with the aim of achieving a serum phosphorus level within the baseline range ±0.5 mg/dl for each patient. If an AE occurred for which a causal relationship with a phosphate binder could not be ruled out, the dose could be reduced. Restricted concomitant medications and therapies were calcium preparations for supplementation, active vitamin D preparations, calcimimetics, and hemodialysis. The dosing regimen of these medications and dialysis conditions, except calcium preparations for supplementation, could not be changed before scheduled assessments at week 9; thereafter, changes in dosing were allowed. Prohibited concomitant medications and therapies were aluminum preparations, niceritrol, nicotinamide, colestimide, cholestyramine, other supplements for phosphorus adsorption, and peritoneal dialysis.

**Table 1:** Baseline characteristics of patients

	Tenapanor (*N* = 67)
Sex	
Female	25 (37.3)
Male	42 (62.7)
Age (yr)	
Mean (SD)	62.7 (10.2)
Median (minimum, maximum)	65.0 (34, 79)
<65	32 (47.8)
≥65	35 (52.2)
Height (cm)	
Mean (SD)	161.3 (9.3)
Weight (kg)	
Day 1 before dialysis, mean (SD)	60.2 (12.7)
Day 1 after dialysis	57.5 (12.4)
Body mass index (kg/m^2^)	
Mean (SD)	22.9 (3.2)
Primary disease	
Diabetic nephropathy	13 (19.4)
Chronic glomerulonephritis	43 (64.2)
Nephrosclerosis	8 (11.9)
Polycystic kidney disease	1 (1.5)
Other	2 (3.0)
Phosphate binders in use at baseline	
Calcium carbonate	26 (38.8)
Sevelamer hydrochloride	51 (76.1)
Lanthanum carbonate	21 (31.3)
Bixalomer	12 (17.9)
Sucroferric oxyhydroxide	7 (10.4)
Ferric citrate hydrate	18 (26.9)
Total phosphate binder tablets per day on day 1	
Mean (SD)	14.7 (6.4)
Total weight of phosphate binder (mg)	
Mean (SD)	5968.0 (2741.6)
Total volume of phosphate binder (mm^3^)	
Mean (SD)	5252.3 (2528.9)
Serum phosphorus level (mg/dl)	
Screening, mean (SD)	5.28 (0.76)
Baseline (Day 1), mean (SD)	5.19 (0.84)

SD, standard deviation.

The values are shown as *n* (%) unless otherwise specified.

### Patients

Male or female patients aged 20 to 79 years undergoing hemodialysis 3 times per week for at least 12 weeks under the same dialysis prescription were enrolled. Patients took ≥2 tablets of phosphate binder 3 times daily and an unchanged dosage of vitamin D or calcimimetic regimen, if any, for 2 weeks before screening. They were required to have serum phosphorus levels ≥3.5 and ≤7.0 mg/dl at screening and during the 3-week observation period.

The main exclusion criteria were intact parathyroid hormone >600 pg/ml; a history of inflammatory bowel disease, diarrhea-predominant irritable bowel syndrome, developed inflammatory bowel disease, or diarrhea-predominant irritable bowel syndrome after pre-enrollment; and the presence of diarrhea or loose stools, defined as Bristol Stool Form Scale score ≥6 and frequency ≥3 for 2 or more days, within 1 week before enrollment.

### Study End Points

The primary end point was the percentage of patients who achieved a ≥30% decrease in the total number of phosphate binder and tenapanor tablets prescribed daily based on an average calculated for week 24 to 26 compared with the number of prescribed phosphate binder tablets at baseline. If patients discontinued after week 11 of the treatment period, an average was calculated based on the 3 most recent time points before discontinuation. In patients who discontinued before week 11 of the treatment period, the outcome was judged as “not achieved.”

The secondary end points were the percentage of patients who achieved a ≥30% decrease in the total number of phosphate binder and tenapanor tablets prescribed daily at each time point after the start of treatment compared with the number of prescribed phosphate binders tablets at baseline; the total number of tablets, the weight and volume of the prescribed daily drugs at each time point after the start of treatment (Supplementary Methods), their change, and percent change from baseline; serum phosphorus levels at each time point after the start of treatment and their change from baseline; and the calcium × phosphorus product and corrected serum calcium level at each time point after the start of treatment and their change from baseline. The exploratory end points were changes from baseline in intact fibroblast growth factor 23 levels, c-terminal fibroblast growth factor 23 levels, intact parathyroid hormone levels, and bone metabolism markers (bone-specific alkaline phosphatase, osteocalcin, procollagen-1 N-terminal propeptide, and tartrate-resistant acid phosphatase-5b). The safety end points were AEs and changes/abnormalities in laboratory values or vital signs.

Data were collected by electronic case report forms. Patient diaries were used to collect the details of defecation status, study drug administration, and phosphate binder treatment.

### Statistical Analysis

The primary analysis set was the modified intent-to-treat analysis set, defined as all patients enrolled in the study, except those who had never received the study drug. The per-protocol analysis set was defined as all patients included in the modified intention-to treat analysis set, except those patients who did not meet the inclusion criteria or patients with major protocol deviations.

Categoric data were summarized using frequencies and percentages, and continuous data were summarized as descriptive statistics. The number of patients, mean, standard deviation (SD), median, minimum, and maximum were calculated for descriptive statistics. The primary end point was tested using a binomial test with a threshold level of 20% and a 1-sided significance level of 0.025. Statistical analyses were performed using SAS version 9.4 (SAS Institute Inc., Cary, NC).

The number of patients required for examining the effect of tenapanor on the reduction in the number of prescribed tablets was evaluated by reference to results from existing clinical trials.^13^ At minimum, 59 patients were required to provide at least 90% power to detect the effect of tenapanor on reducing the number of prescribed tablets using a binomial test with a 1-sided significance level of 0.025. Thus, the target sample size was set at 60 patients.

### Ethical Considerations

The institutional review boards of the participating sites approved the study protocol and associated documents. The study procedures were compliant with the Declaration of Helsinki and Good Clinical Practice Guidelines, as well as local regulations. Patient confidentiality was maintained throughout. All patients provided informed consent for study participation before the initiation of study procedures and could withdraw consent at any time during the study.

## Results

### Patient Disposition, Baseline Characteristics, and Dosing

**Figure 2:**
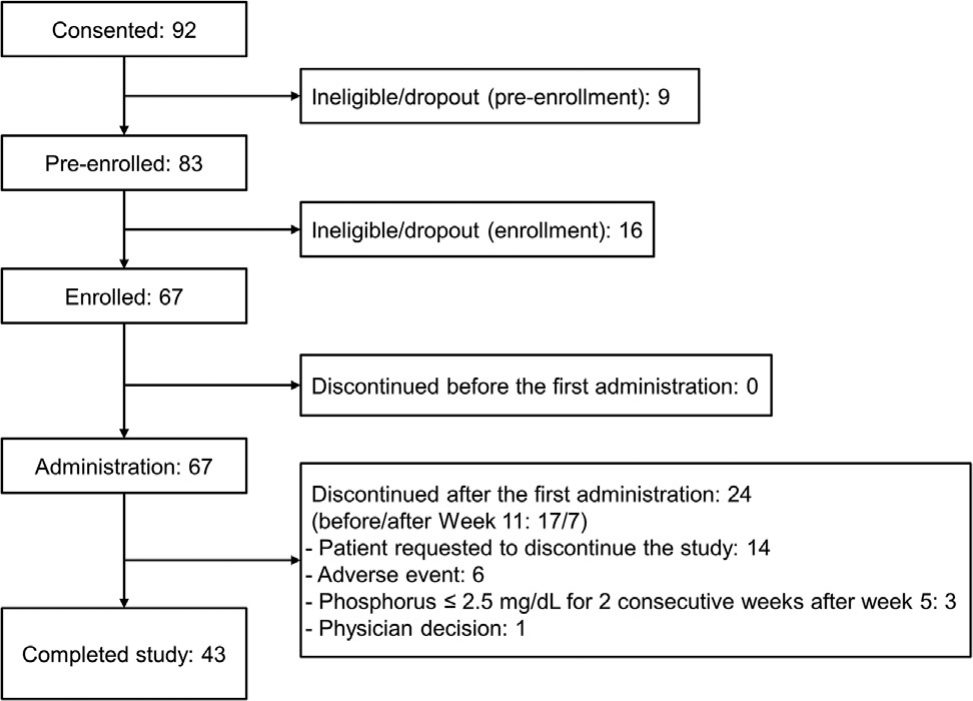
Patient disposition.

Of the 92 patients who consented to participate, a total of 67 patients were enrolled in the study and received at least 1 administration of tenapanor. Twenty-four patients (35.8%) discontinued the study; the reasons included patient request to discontinue the study (*n* = 14), AE (*n* = 6), phosphorus ≤2.5 mg/dl for 2 consecutive weeks after week 5 (*n* = 3), and physician decision (*n* = 1). Forty-three patients (64.2%) completed the study (Figure 2).

Most patients were male (62.7%) and had a median (minimum, maximum) age of 65.0 (34, 79) years. Chronic glomerulonephritis was the most common primary disease (64.2%) followed by diabetic nephropathy (19.4%) and nephrosclerosis (11.9%). At baseline, the 3 most common phosphate binders used were sevelamer hydrochloride (76.1%), calcium carbonate (38.8%), and lanthanum carbonate (31.3%). The mean (SD) serum phosphorus level at the screening visit was 5.28 (0.76) mg/dl (Table 1).

**Figure 3:**
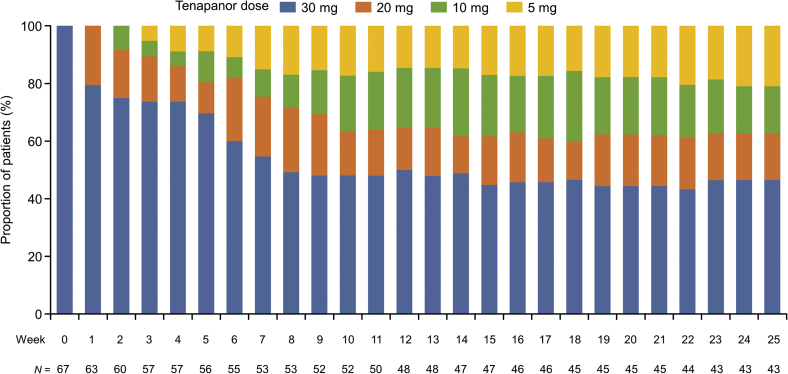
The proportion of patients in each dose category of tenapanor.

By week 8, nearly half of the patients were receiving a tenapanor 30-mg dose, with the remainder receiving either a 20-, 10-, or 5-mg dose. From week 8 and on, the tenapanor dose remained constant until the end of the study (Figure 3).

### Primary End Point

Compared with the number of prescribed daily phosphate binder tablets at baseline, 71.6% of patients achieved the primary end point, which was a ≥30% decrease in the mean total daily phosphate binder and tenapanor tablets (95% confidence interval, 59.3–82.0; *P* < 0.001). In addition, 52.2% and 28.4% of patients achieved a ≥50% decrease in the mean total daily phosphate binder and tenapanor tablets and completely switched from phosphate binder tablets (95% confidence interval, 39.7–64.6 and 18.0–40.7; *P* < 0.001 for both) (Table 2).

**Table 2:** Dose reduction status of phosphate binder by investigational drug dose (or achievement status of primary end point)

End Points	Tenapanor dose^a^				
	Total (*N* = 67)	30 mg (*N* = 29)	20 mg (*N* = 11)	10 mg (*N* = 8)	5 mg (*N* = 19)
Achieved ≥30% reduction in tenapanor + phosphate binder tablets	48 (71.6) (59.3–82.0) <0.001	19 (65.5) (45.7–82.1) <0.001	7 (63.6) (30.8–89.1) <0.001	7 (87.5) (47.3–99.7) <0.001	15 (78.9) (54.4–93.9) <0.001
Achieved ≥50% reduction in tenapanor + phosphate binder tablets	35 (52.2) (39.7–64.6) <0.001	15 (51.7) (32.5–70.6) <0.001	6 (54.5) (23.4–83.3) <0.001	4 (50.0) (15.7–84.3) <0.001	10 (52.6) (28.9–75.6) <0.001
Achieved complete switching to tenapanor from phosphate binder tablets	19 (28.4) (18.0–40.7) <0.001	8 (27.6) (12.7–47.2) <0.001	3 (27.3) (6.0–61.0) 0.002	2 (25.0) (3.2–65.1) 0.016	6 (31.6) (12.6–56.6) < 0.001

Data are presented as *n* (%), 95% confidence interval (minimum–maximum), and *P* value.

a The dose of tenapanor reported in the table is the final dose being administered at week 26 for each patient who completed the study or the dose of tenapanor that was being administered at the time of discontinuation for patients who discontinued before week 26.

### Secondary End Points

**Figure 4:**
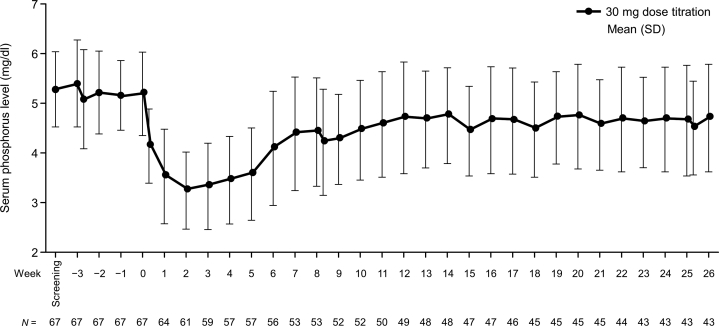
The mean changes (± standard deviation) in serum phosphorus over time up to week 26. SD, standard deviation.

**Figure 5:**
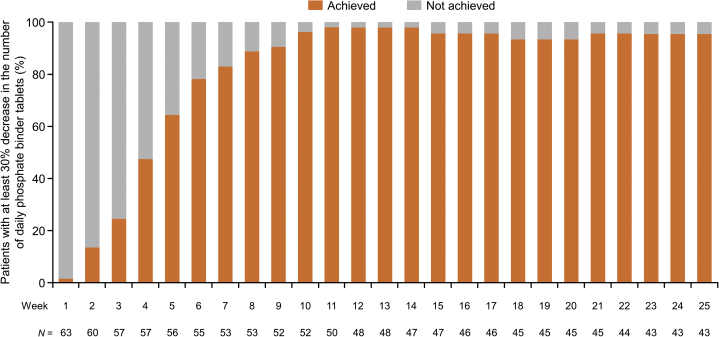
The proportion of patients who achieved at least a 30% decrease in the number of daily phosphate binder tablets (%).

**Figure 6:**
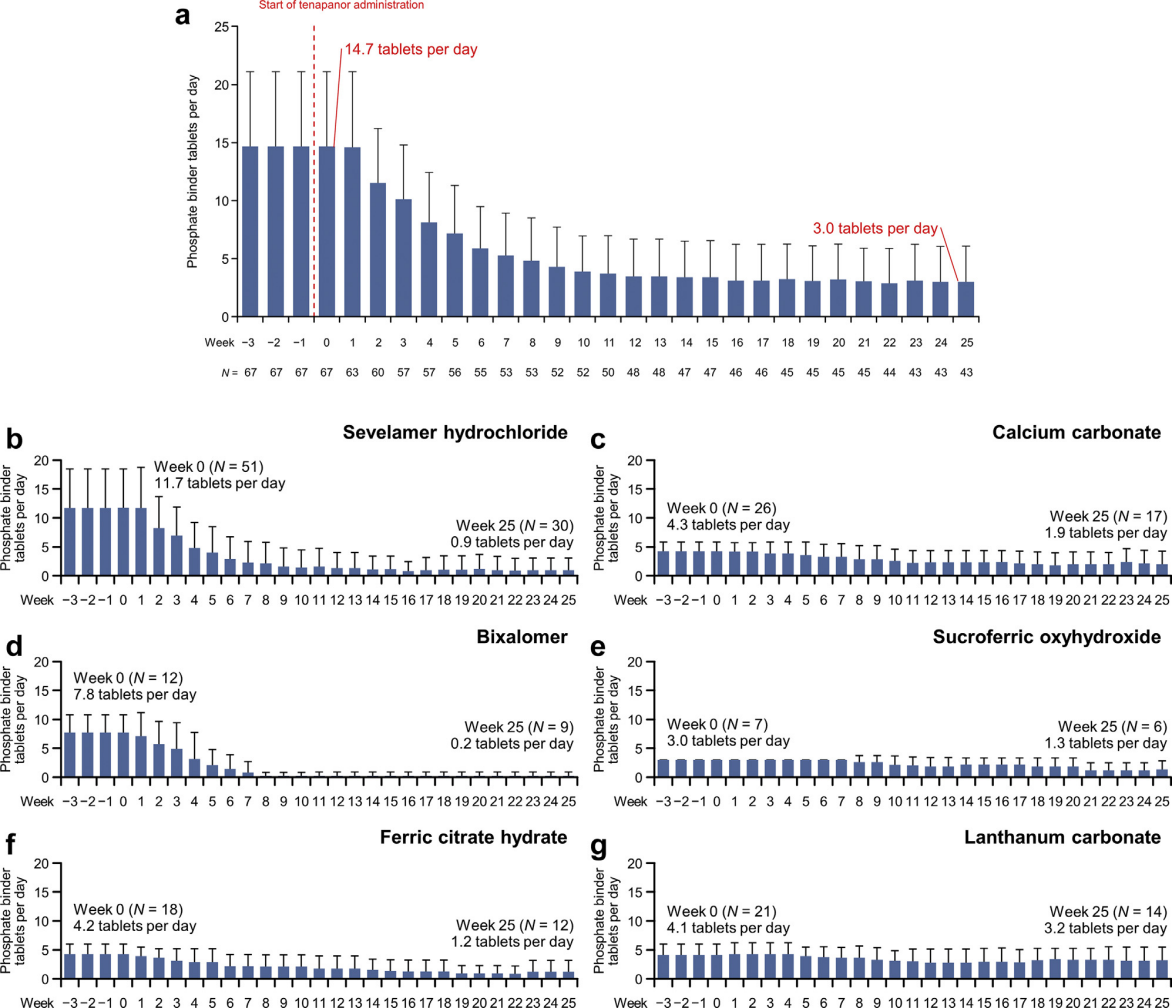
The administration of phosphate binder tablets. (a) The mean number of tablets administered per day and (b–g) the mean number of phosphate binder tablets administered per day by type of phosphate binder.

At week 26, the mean (SD) phosphorus level was 4.71 (1.08) mg/dl, and the mean change from baseline was −0.40 (1.15) mg/dl (Figure 4). As early as week 5, 36 (64.3%) patients achieved a 30% reduction in the number of daily phosphate binder tablets (Figure 5). A marked decrease in the mean (SD) number of daily phosphate binder tablets was observed from week 0 (14.7 [6.4]) to week 10 of treatment (to ~4 tablets), and the number of tablets remained almost constant until the end of treatment. At week 26, the mean (SD) total number of tablets of phosphate binder decreased to 3.0 (3.1) tablets (Figure 6a), with a mean change of −12.1 (6.4) tablets per day and a mean percent change from baseline of −80.4% (19.7%). The number of tablets decreased for all types of phosphate binders. Among these, the mean number of bixalomer, sevelamer hydrochloride, and ferric citrate hydrate tablets decreased remarkably (Figures 6b–g).

The mean (SD) total weight of the prescribed daily tablets of phosphate binder at baseline was 5968.0 (2741.6) mg. The tablet weight decreased weekly, beginning with the first administration of tenapanor and reaching a total weight of 1773.4 (2102.5) mg at week 26. The total weight of the prescribed daily tablets of phosphate binder and tenapanor combined at week 26 was 2183.2 (2098.5) mg, with a mean change of −4138.0 (2570.3) mg and a mean percent change from baseline of −66.8% (24.6%).

The mean (SD) total volume of the prescribed daily tablets of phosphate binder at baseline was 5252.3 (2528.9) mm^3^. The tablet volume subsequently decreased each week, reaching 1110.4 (1262.4) mm^3^ at week 26. The total volume of prescribed tablets combined (phosphate binder and tenapanor) was 1448.6 (1263.9) mm^3^ at week 26, with a mean change and mean percent change from baseline of −4134.8 (2673.5) mm^3^ and −72.8% (20.1%), respectively.

The mean (SD) calcium × phosphorus product was 47.6 (7.7) mg/dl^2^ at baseline and 43.7 (10.0) mg/dl^2^ at week 26, with a mean change from baseline of −3.1 (10.5) mg/dl^2^. There was no remarkable change in the mean corrected calcium levels and calcium × phosphorus product during the study. The mean (SD) corrected calcium levels at baseline and week 26 were 9.2 (0.6) mg/dl and 9.3 (0.5) mg/dl, respectively. The levels remained relatively constant throughout the study, with a mean change from baseline of 0.1 (0.4) mg/dl.

### Exploratory Analysis

At baseline, the mean (SD) intact fibroblast growth factor 23 level was 6543.4 (7700.7) pg/ml, and at week 26 it was 4720.9 (6422.4) pg/ml, with a mean (SD) change from baseline of −1334.5 (3676.1) pg/ml. At baseline, the mean (SD) c-terminal fibroblast growth factor 23 level was 5644.6 (5767.7) RU/ml, and at week 26, it was 4851.3 (5585.0) RU/ml, with a mean (SD) change from baseline of 200.4 (3771.7) RU/ml. The intact parathyroid hormone level at baseline was 112.9 (66.8) pg/ml, and at week 26, it was 127.0 (93.1) pg/ml, with a mean change from baseline of 14.0 (62.6) pg/ml. Mean bone turnover markers, namely bone-specific alkaline phosphatase, osteocalcin, procollagen-1 N-terminal propeptide, and tartrate-resistant acid phosphatase-5b, remained fairly unchanged over the 26-week treatment period.

### Safety

The incidence of any AE was 92.5% (62/67) and that of serious AEs was 7.5% (5/67). Among the AEs with an incidence above 5%, the most common AE was diarrhea (76.1% [51/67]) (Table 3). Serious AEs were acute myocardial infarction, diarrhea, vascular puncture site pain, diverticulitis, and cerebral infarction (1 patient each [1.5%]).

**Table 3:** Summary of any AEs and drug-related with an incidence above 5%

	Tenapanor (*N* = 67)	
	AEs	Drug-related AEs
Patients with any AE	62 (92.5)	51 (76.1)
Serious	5 (7.5)	2 (3.0)
Death	0 (0.0)	0 (0.0)
Other serious AEs	5 (7.5)	2 (3.0)
Other significant AEs^a^	38 (56.7)	38 (56.7)
Study medication discontinuation	6 (9.0)	4 (6.0)
Patients with any AE (system organ class)		
Preferred term		
(Gastrointestinal disorders)	53 (79.1)	51 (76.1)
Diarrhea	51 (76.1)	50 (74.6)
(Infections and infestations)	19 (28.4)	—
Nasopharyngitis	9 (13.4)	—

AE, adverse event.

The values are shown as *n* (%).

a Other significant adverse events were defined as “all non-serious adverse events that resulted in discontinuation, suspension, or dose reduction of study drug administration.”

The incidence of any drug-related AEs was 76.1% (51/67), and the most common drug-related AEs were gastrointestinal disorders (76.1%), predominantly diarrhea (74.6%) (Table 3). By diarrhea severity, 31 patients had mild diarrhea, 19 had moderate diarrhea, and no patients had severe diarrhea. The incidence of serious drug-related AEs was 3.0% (2/67, 1 patient with myocardial infarction and 1 with diarrhea). The patient with myocardial infarction discontinued tenapanor 30-mg treatment, underwent percutaneous coronary intervention, and recovered. The patient with diarrhea had a dose reduction to 5 mg and discontinued treatment.

There were no clinically relevant changes/abnormalities in vital signs and blood chemistry and hematology laboratory values. The mean (SD) change from baseline to week 26 was −0.1 (2.5) mEq/l in sodium, 0.1 (0.6) mEq/l in potassium, −0.2 (3.3) mEq/l in chlorine, −0.3 (0.4) mg/dl in magnesium, and 0.2 (2.0) mmol/l in bicarbonate. No clinically relevant changes/abnormalities were observed in electrocardiographic parameters, except in 1 patient who presented acute myocardial infarction (detailed previously). No deaths occurred during the study.

**Figure 7:**
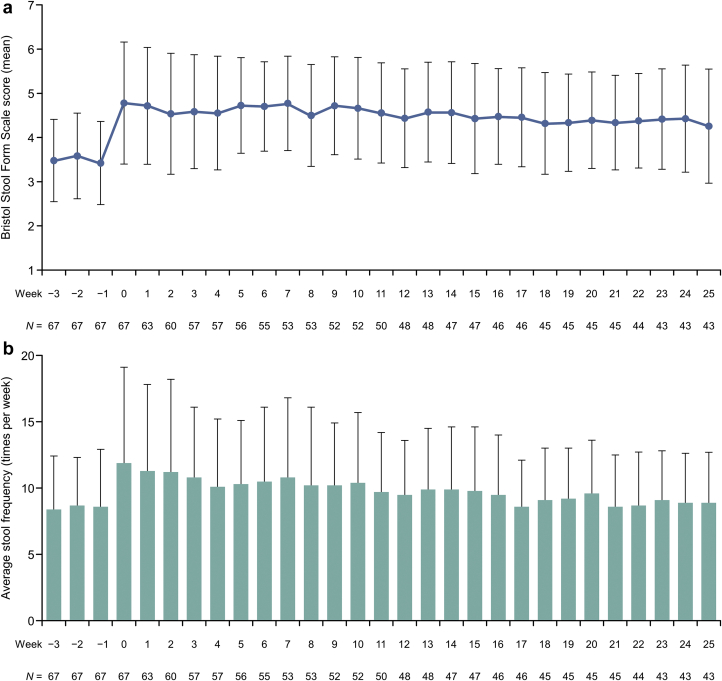
(a) The Bristol Stool Form Scale score and (b) average stool frequency during the study.

The Bristol Stool Form Scale score increased after tenapanor administration and then decreased slightly throughout the study (Figure 7a). The number of bowel movements increased after administration but decreased as the study progressed and then returned near baseline value during week 26 (Figure 7b).

## Discussion

In this first phase 2 study of tenapanor for Japanese hemodialysis patients taking phosphate binders, tenapanor reduced the phosphate binder pill burden. The primary end point, a ≥30% decrease in the mean total daily phosphate binder and tenapanor tablets, was met by over 70% of patients. Of note, over 60% of patients achieved the primary end point as early as week 5 after beginning tenapanor administration. The total mean number of daily tablets of phosphate binder was decreased from 14.7 tablets to 3.0 tablets. The number of tablets decreased overall, and this decrease was more marked for bixalomer, sevelamer hydrochloride, and ferric citrate hydrate. A reason for this might be that the investigator may have tended to decrease the dose of bixalomer, sevelamer hydrochloride, and ferric citrate hydrate, which were high-dose medications first. As such, although phosphate binders were frequently used in combination with multiple drugs, it was possible that the doses of other drugs were decreased after decreasing bixalomer, sevelamer hydrochloride, and ferric citrate hydrate. Furthermore, because the average number of calcium carbonate, sucroferric oxyhydroxide, and lanthanum carbonate tablets was originally low at baseline, this may have limited the extent to which the mean number of tablets of these phosphate binders could be reduced. However, there was a reduction in the average number of tablets of calcium carbonate, sucroferric oxyhydroxide, and lanthanum carbonate. Further study is needed to clarify these findings.

After the start of tenapanor administration, the serum phosphorus level was reduced by week 2 and was relatively well controlled by week 26, with a mean change from baseline of −0.40 mg/dl at week 26. According to the current study design, once the serum phosphorus level decreased with tenapanor administration, the phosphate binder dose was reduced, making it difficult to evaluate the serum phosphorus-lowering efficacy of tenapanor throughout the study period. However, the serum phosphorus level decreased by 1.88 mg/dl from baseline to week 2, which is likely not affected by phosphate binder dose reduction in this study.

Patients who completely switched from their phosphate binder regimen accounted for about 30% of the patients in this study. Tenapanor has a small tablet diameter,^13^ and a single tablet is taken twice daily, whereas phosphate binders are taken 3 times daily. Patients who completely switched from the phosphate binder regimen were able to reduce not only the number of tablets taken but also the number of medication administration times per day for hyperphosphatemia. Notably, some patients in whom the tenapanor dose was reduced to 5 mg were also able to completely stop their phosphate binder regimen, confirming the efficacy of tenapanor treatment.

Presently, the serum phosphorus-lowering effect of tenapanor was observed even in patients who were concomitantly receiving phosphate binders and whose serum phosphorus concentration was controlled to a level between 3.5 and 7.0 mg/dl. Tenapanor inhibits the sodium/hydrogen exchanger isoform 3 transporter, which suppresses phosphorus absorption in the intestinal tract; therefore, phosphorus accumulates in the intestinal tract, where it may bind more easily to the phosphate binder therein.

The incidence of diarrhea in this study was approximately 75% with a starting dose of tenapanor of 30 mg; however, only 4 (6.0%) patients discontinued the study because of diarrhea as an AE. Of these 4 patients, 3 patients improved or recovered. Therefore, tenapanor was considered to be generally well tolerated over the dosing period of 6 months. A separate phase 2 monotherapy dose-response study was conducted in parallel with this study (M. Inaba *et al.*, unpublished data, 2021). Based on the results of that study, we plan to decide the starting dose of tenapanor in future phase 3 studies. In addition, the planned phase 3 long-term safety study will assess the safety of tenapanor administration for 1 year. Thus, we will continue to confirm the tolerability related to diarrhea during the long-term administration of tenapanor.

In this study, given that several patients discontinued the study during the initial weeks because of diarrhea, it is important to control the symptoms of diarrhea at an early treatment stage. Hemodialysis patients originally have constipation and are often prescribed laxatives. Some patients discontinued the prescription of laxatives because they developed diarrhea after the administration of tenapanor. Because tenapanor can cause soft stools, it is possible that discontinuing laxative use when starting tenapanor treatment may help manage symptoms of diarrhea.

Existing phosphate binders may lead to secondary effects, such as replenishment of calcium with calcium carbonate, replenishment of iron with iron-based phosphate binders, and a reduction of low-density lipoprotein cholesterol with sevelamer.^8^ As a result of adding on tenapanor and switching from the prescribed phosphate binder regimen, drug-related AEs associated with a decrease in the secondary effects of phosphate binders were not observed. Thus, it is expected that patients can safely switch from phosphate binders to tenapanor in clinical practice.

The major limitation of this study was that the target population consisted of patients receiving many phosphate binder tablets at the time of registration, including many patients taking polymeric phosphate binders. Therefore, it is likely that this study enrolled a high proportion of patients who were taking large doses of sevelamer or bixalomer and who likely had a relatively high pill burden. We considered that reducing the number of tablets was causally linked to a decrease in the drug load.

In conclusion, by adding tenapanor to existing phosphate binder regimens, patients could reduce not only the phosphate binder pill burden, but also some successfully switched from the prescribed phosphate binder regimen completely. Adding tenapanor and reducing the amount of phosphate binder did not impede the control of the serum phosphorus levels, even in patients who completely switched to tenapanor from their phosphate binder regimen. The serum phosphorus level at week 26 was well managed. Even though many patients experienced diarrhea, few discontinued the study because of diarrhea over the 6-month dosing period. For more widespread use of tenapanor, an increased understanding of the optimal management of drug-related diarrhea will be necessary. The present findings suggest that tenapanor may reduce the pill burden of phosphate binders among patients on phosphate binder regimens or eliminate the need for phosphate binders while effectively controlling phosphorus.

## Disclosure

TA has received personal fees from Kyowa Kirin Co., Ltd., during the conduct of the study and personal fees from Astellas, Bayer Yakuhin Ltd., Kissei Pharmaceutical Co. Ltd., Ono Pharmaceutical Co. Ltd., Fuso Pharmaceutical Industries Ltd., Torii Pharmaceutical Co. Ltd., GlaxoSmithKline, JT Pharmaceuticals, Nipro Corporation, Otsuka, Sanwa Chemical, and Chugai Pharmaceutical Co. Ltd. outside the submitted work. MF has received personal fees from Kyowa Kirin Co., Ltd., Ono Pharmaceutical Co. Ltd., Torii Pharmaceutical Co. Ltd., and Kissei Pharmaceutical Co. Ltd. and grants from Bayer Yakuhin Ltd., during the conduct of the study. YS, KI, and HK are employees of Kyowa Kirin Co., Ltd.
