# Supplementary material for: Effect of Tenapanor on Phosphate Binder Pill Burden in Hemodialysis Patients
Source: Kidney Int Rep. 2021 Jul 8;6(9):2371–80. doi: 10.1016/j.ekir.2021.06.030 (PMC8418975; doi:10.1016/j.ekir.2021.06.030)
Supplement: Supplementary File (PDF) [file mmc1.pdf]

SUPPLEMENTARY METHODS

Calculations of tablet weight and volume

The weights of tablets of tenapanor and ferric citrate hydrate were calculated by measuring tenapanor and commercial ferric citrate hydrate at our CMC Research Center. Other phosphate binders were described by citing the information on the respective package insert. In addition, the volume was calculated by measuring tenapanor and all phosphate binder tablet types at our CMC Research Center using tenapanor and commercial phosphate binders. Of note, the volume of granules could not be measured. For granules, we used the following calculations:

a) Precipitated calcium carbonate, fine granules 500 mg (1 packet) = 1 tablet

   1000 mg (1 packet) = 2 tablets.

b) Lanthanum carbonate hydrate, granules 250 mg (1 packet) = 1 tablet

   500 mg (1 packet) = 1 tablet.

c) Bixalomer, granules 250 mg (290 mg by weight) = 1 tablet

d) Sucroferric oxyhydroxide, granules 250 mg (1 packet) = 1 tablet

   500 mg (1 packet) = 1 tablet
